# Genetic Evolution Analysis and Host Characteristics of Hantavirus in Yunnan Province, China

**DOI:** 10.3390/ijerph192013433

**Published:** 2022-10-18

**Authors:** Na Wang, Jia-Xiang Yin, Yao Zhang, Li Wu, Wen-Hong Li, Yun-Yan Luo, Rui Li, Zi-Wei Li, Shu-Qing Liu

**Affiliations:** School of Public Health, Dali University, Dali 671000, China

**Keywords:** hantavirus, hemorrhagic fever with renal syndrome (HFRS), genetic diversity, host

## Abstract

For a long time, the epidemic situation of hemorrhagic fever with renal syndrome (HFRS) caused by hantavirus (HV) in Yunnan Province of China has been relatively severe. The molecular epidemiology and host characteristics of HV in Yunnan Province are still not completely clear, and the systematic and long-term investigation of the epidemic area is very limited. In this study, a total of 488 murine-shaped animals were captured in the three regions of Mile City, Mangshi City and Lianghe County in Yunnan Province, and then the type of HV was identified by multiplex real-time RT-PCR and sequenced. The results indicate that 2.46% of the murine-shaped animal specimens were infected with HV. A new subtype of Seoul virus (SEOV) was found in the rare rat species *Rattus nitidus* in Lianghe County, and the two strains of this new subtype were named YNLH-K40 and YNLH-K53. Through the phylogenetic analysis of this new subtype, it is shown that this new subtype is very similar to the type S5 of SEOV, which is previously described as the main cause for the high incidence of HFRS in Longquan City, Zhejiang Province, China. This new subtype is highly likely to cause human infection and disease. Therefore, in addition to further promoting the improvement of the HV gene database and strengthening the discovery and monitoring of the host animals in Yunnan Province, more attention should be paid to the pathogenic potential of the newly discovered HV type.

## 1. Introduction

Hantavirus (HV) is an enveloped, single stranded, negative sense RNA virus and belongs to the genus *Orthohantavirus*, family *Hantaviridae*, order *Bunyavirales* [1]. Two fatal acute infectious diseases of hemorrhagic fever with renal syndrome (HFRS) and hantavirus cardiopulmonary syndrome (HCPS) can be caused by HV [2]. The genotype of HV is very abundant. According to current statistics, more than 50 kinds of HV have been confirmed, of which at least 28 kinds can cause human diseases [3]. Humans are mainly infected by the inhalation of aerosol formed by the excreta and secretion of rodent host animals, as well as wound contact. Meanwhile, the ingestion of water, soil or food contaminated by the excreta and secretion of host animals can also cause human infection [4]. Annually, more than 15,000 cases caused by HV are reported in the world [5]. HCPS is mainly prevalent in the American continent, and the early case fatality rate was once as high as 50% [6]. HFRS is primarily prevalent in Eurasia, with an early case fatality rate of 12% [7]. It is noteworthy that about 90% of the global HFRS cases are reported in China [8]. From 2016 to 2018, China reported about 11,000 HFRS cases every year [9]. Even today, HFRS infection is still a serious public health problem in China [10]. To date, it has been confirmed that the HFRS infection is associated with at least seven different types of HV [11]. In China and even all of Asia, it is mainly caused by Seoul virus (SEOV) and Hantaan virus (HTNV), which frequently cause the human epidemic of HFRS.

Unlike other viruses of the order *Bunyavirales*, HV is the only one that is not transmitted through arthropod vectors but mainly by *rodentia* [12]. Subsequently, HV was discovered in bats (*Chiroptera*) and *Soricomorpha* [13]. In addition, the finding of HV in some domestic animals has also been reported, such as cats, dogs, cattle, sheep, pigs and rabbits, which further expands the host range of HV [14]. The changes in economic and environmental factors have also made human contact with hantavirus hosts more and more close. HV type is closely related to selective host conversion and regional adaptation and constantly evolves in the form of gene recombination. Accordingly, the generation of highly pathogenic and highly infectious new virus strains which can bring new challenges to disease prevention and control cannot be ruled out [14]. At present, there is no FDA-approved HV vaccine or an effective treatment strategy and antiviral plan for HFRS and HCPS [5,15]. Expectedly, the number of HFRS cases will constantly increase for some period [16].

Yunnan Province, one of the southernmost provinces in the Chinese Mainland, is connected with Southeast Asian countries such as Myanmar, Vietnam and Laos by mountains and rivers. It vigorously develops border trade and domestic and foreign tourism and is the cooperation and exchange center between China and Southeast Asia. Meanwhile, Yunnan Province is also the region with the richest species diversity in the Asian continent [17]. The unique tropical to subtropical climate of Yunnan Province offers sufficient living conditions for murine species, which is conducive to the spread of pathogens of murine transmissible diseases such as HV. HFRS cases are reported every year in Yunnan Province. Moreover, due to the special geographical location and trade and tourism status of Yunnan Province, the mobility of the population and the contact opportunities of the population with the virus host and disease transmission media evidently increase. This situation enhances the risk of population exposure and the risk of the population contracting diseases to a certain extent, thereby posing a threat to human life and health. Thereby, for better preventing and controlling of HV, it is very significant to continuously carry out the epidemiological and pathogenic monitoring of HV in Yunnan Province.

## 2. Materials and Methods

### 2.1. Ethics Statement

This Experimental Protocol was reviewed by the Medical Ethics Committee of Dali University and conforms to the principles of medical experiment ethics and the relevant provisions of the national medical experiment ethics and welfare. Endangered or protected animal species were not included in this research.

### 2.2. Sample Collection

**Figure 1:**
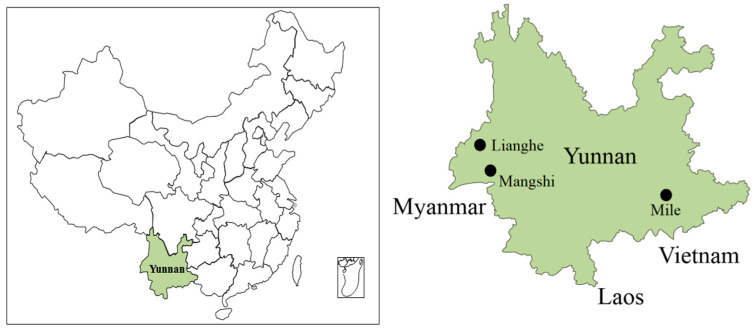
Specimen trapping sites in Yunnan Province, China.

In this study, the murine-shaped animal specimens were captured in Mile City, Mangshi City and Lianghe County in Yunnan Province through the night clamping method in July and August 2019. The specimen trapping sites are shown in Figure 1. The trapping sites are village settlements and the surrounding fields. The murine species were identified by morphology, and the murine lungs were collected using aseptic procedures. After sampling, the specimens were stored in liquid nitrogen and transported to Dali University with dry ice at low temperature.

### 2.3. Extraction of Nucleic Acid and HV Detection

Tissue total RNA extraction kit (Bioteke, Beijing, China) was adopted for extracting the total RNA from 50–100 mg lung tissue. The TaqMan^®^ Fast Virus 1-Step Master Mix (Applied Biosystems, Waltham, WA, USA) was used to detect and type nucleic acids. HTNV- and SEOV-specific primers used were referred to China Center for Disease Control and Prevention [18]. HTNV forward primer: 5′-GCT TCT TCC AGA TAC AGC AG-3′, reverse primer: 5′-GCC TTT GAC TCC TTT GTC TCC AT-3′, probe: 5′-CCT GCA ACA AAC AGG GAY TAC TTA CGG CA-3′. SEOV forward primer: 5′-GAT GAA CTG AAG CGC CAA CTT-3′, reverse primer: 5′-CCC TGT AGG ATC CCG GTC TT-3′, probe: 5′-CCG ACA GGA TTG CAG CAG GGA AGA A-3′. HTNV was input into FAM channel, and SEOV was input into VIC channel. The real-time RT-PCR reaction was performed at the following conditions: ① 50 °C 5 min; ② 95 °C 20 s; ③ 95 °C 3 s, 60 ℃ 30 s, 40 cycles in total. Samples with CT values ≤ 35 and smooth amplification curves were considered as HV positive.

### 2.4. Amplification and Sequencing of S-Segment Gene of SEOV

Samples confirmed to be positive by real-time RT-PCR were reverse transcribed into cDNA with SuperScript^®^ IV First-Strand cDNA Synthesis Reaction (Invitrogen, Waltham, MA, USA). Reverse transcription primer P14 was derived from China Center for Disease Control and Prevention. The following 2 pairs of S-segment primers were synthesized [17]: S1F: 5′-GCT GAA GAG ATA ACA CCT GGA AGA TTC CG-3′, S1R: 5′-ATC TGA GCC AGG CTA CGA AGC-3′; S2F: 5′-AAT TGT TAT GTT TAT GGT TGC CTG GG-3′, S2R: 5′-TAG TAG TAT GCT CCC TAA AAA GAC AAA AGG-3′. PCR amplification was conducted using DreamTaq Green PCR Master Mix (2X) (Thermo Scientific, Waltham, WA, USA). The 2% agarose gel electrophoresis was applied to detect the PCR amplification products, and the target products were sent to Sangong Bioengineering (Shanghai, China) Co., Ltd. for sequencing.

### 2.5. Genetic Characterization and Phylogenetic Analysis

Other sequences used in this study were HV genome sequences that were submitted in GenBank. The National Center for Biotechnology Information (NCBI) BLAST and DNAstar software were used to analyze the homology and genetic evolution characteristics of HV and the similarity and variation in nucleotide and amino acid of HV sequences newly discovered in Yunnan Province and other HV sequences. The phylogenetic tree was constructed using the neighbor-joining (NJ) method based on the principle of minimum evolution through MEGA-X software. The bootstrap analysis of 1000 iterations was used to estimate the node support. The clades were displayed with the bootstrap values ≥ 95%.

### 2.6. Nucleotide Sequence Accession Numbers

The partial S-segment sequences for HV obtained in this research were submitted to GenBank with accession numbers of OP381186-OP381187. Other sequences employed in this research were HV genome sequences that were submitted in GenBank.

## 3. Results

### 3.1. HV Infection in Murine-Shaped Animals

A total of 488 murine-shaped animals were captured, including 16 murine species. The dominant species *Rattus tanezumi* had 244 specimens, making up 50.00% of the total, and the dominant species *Suncus murinus* had 150 specimens, making up 30.74% of the total. The other murine species in the total specimens were not more than 2.87%. The total RNA of lung tissue was screened by double real-time RT-PCR to detect HV nucleic acid. A total of 12 nucleic acid positive specimens were identified, all of which belong to Seoul virus (SEOV), with a detection rate of 2.46%, including three murine species. Among these obtained positive specimens, the *Rattus tanezumi*, *Suncus murinus* and *Rattus nitidus* account for 66.67%, 16.67% and 16.67%, respectively. The detection rate of 3.45% for Lianghe County is the highest, followed by 1.58% for Mangshi City and 1.52% for Mile City. In the detected specimens with HV, the dominant species *Rattus tanezumi* accounts for an absolute proportion, and HV was also detected in *Rattus nitidus*, which does not belong to the dominant species. The numbers for trapped murine-shaped animals and HV-positive murine-shaped animals in three regions are given in Table 1.

### 3.2. Sequence Homology Analysis

The S-segment gene of HV was obtained from two murine lung specimens with positive nucleic acid detection and sequenced. The nucleotide and amino acid homologies of newly sequenced strains YNLH-K40 and YNLH-K53 with the representative six subtypes of SEOV and the other reference strains such as HTNV, DOBV, SNV, PUUV and ANDV in GenBank were compared by DNAstar software. The comparison results are presented in Table 2. Results reveal that the nucleic acid homologies of the newly sequenced strains with SEOV are significantly higher than the nucleic acid homologies of the newly sequenced strains with other types. The nucleic acid homology and amino acid homology between the newly sequenced strains and SEOV are 84.0–88.5% and 83.6–87.8%, respectively. The nucleic acid homologies of the newly sequenced strains with HTNV76-118 and PUUVP360 strains are only 65.7–66.3% and 40.6–41.0%, respectively. The sequence homology of the specimens between murine species for the S-segment sequences YNLH-K40 and YNLH-K53 is 99.3%, and the gene variation is not obvious, belonging to the same subtype. Previous studies showed that the S1 and S3 subtypes of Seoul virus are mainly prevalent in Yunnan Province [17]. By comparing the newly sequenced strains with the reference strains R22 (S1 subtype) and ZT10 (S3 subtype), it is found that the homologies are only 85.9–86.0% and 84.0–84.3%, respectively. Consequently, the newly sequenced strains do not belong to the same subtype as S1 and S3. In comparison with other subtypes, the nucleotide sequence of the newly sequenced strains has the highest homology (88.5%) with the S5 subtype strain LongquanRn-09-132 and the lowest homology (84.0%) with the S3 subtype strain ZT10.

### 3.3. Genetic Evolution Analysis

**Figure 2:**
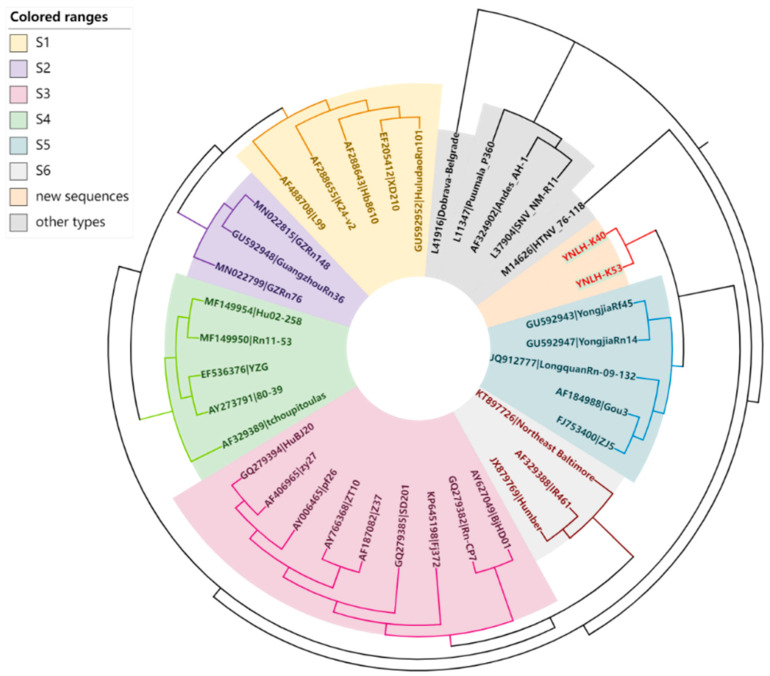
Phylogenetic tree constructed using nucleotide sequences of the Seoul virus partial S-segment gained from Yunnan Province, China. S1 to S6 are the six subtypes of SEOV, respectively, and the gray part covers the five other types of HV of HTNV, PUUV, SUV, ANDV and DOBV.

**Figure 3:**
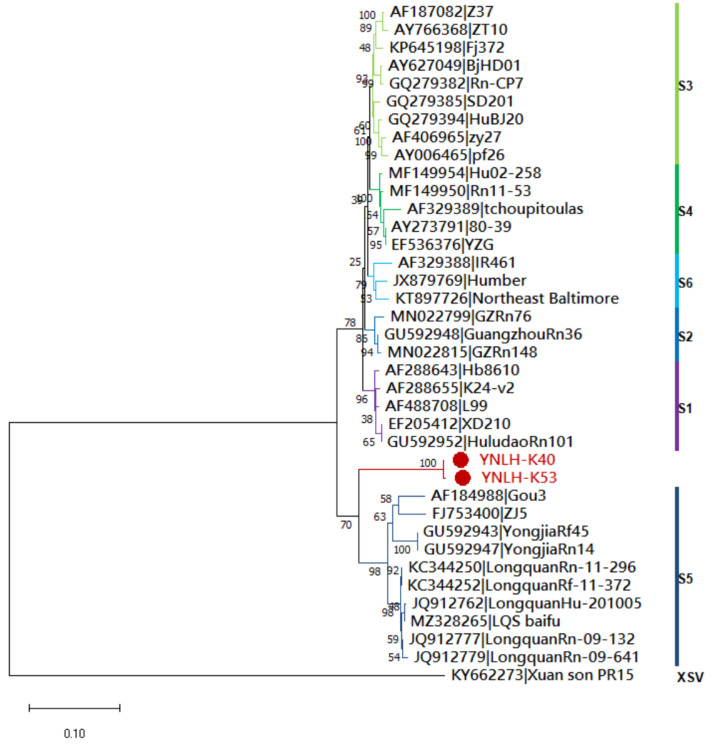
Phylogenetic tree constructed using nucleotide sequences of the Seoul virus partial S-segment gained from Yunnan Province, China. Thirty-five representative Orthohantavirus sequences were included for comparison, and one Xuan son virus sequence was used as the outgroup. Note: the red circles represent the newly discovered HV sequences in this study.

The S-segment gene sequences YNLN-K40 and YNLH-K53 of HV obtained in this study were analyzed by genetic evolution with the reference sequences of each gene subtype of SEOV and the sequences of the representative strains of HTNV, DOBV, SNV, PUUV and ANDV. The corresponding phylogenetic tree was constructed and is shown in Figure 2. The results illustrate that the viral gene sequences obtained in this study cluster in the clade to which the SEOV gene sequence belongs and are far from the reference strains such as DOBV, HTNV, SNV, PUUV and ANDV. YNLH-K40 and YNLH-K53 are clustered to form an independent branch, which is closest to the S5 subtype branch composed of GOU3, ZJ5, LongquanRn09-132 and other strains originating from Longquan City, Zhejiang Province, China, but the highest nucleotide homology between these sequences is only 88.5%. Meanwhile, the BLAST analysis results of NCBI suggest that the highest homology of YNLH-K40 and YNLH-K53 with the published sequences is less than 90%. Hence, according to the recommendations of the International Committee on virological nomenclature on the typing of HV, it can be considered that YNLH-K40 and YNLH-K53 belong to the new gene subtype of SEOV. It is worth noting that YNLH-K40 and YNLH-K53 are closely associated with the previously described GOUV variants, but they are in a separate evolutionary sub-branch, as shown in Figure 3. This phylogenetic pattern indicates the new genetic variation in the newly sequenced strains, and the epidemic strains have regional characteristics.

## 4. Discussion

Humans generally lack immunity to HV. Once human beings are infected, HV will pose a great threat to human safety and even life and health. China is the country most seriously affected by HV in the world [14]. Except Qinghai Province and Xinjiang Province in China, the other thirty-one provinces, cities and autonomous regions in China all have reported HFRS cases. Taiwan has also reported cases of HV infection. Meanwhile, the epidemic foci of HFRS in China are expanding continuously, showing the tread of moving from north to south and from rural areas to urban centers [19]. The distribution features of the epidemic in China are still highly sporadic and relatively concentrated, and the situation is not optimistic. As a result, it is very important for the prevention and control of HFRS to systematically implement the epidemiological and pathogenic surveillance of HFRS in all provinces of China.

Yunnan Province is one of the provinces with the most serious epidemic of natural focal disease in China, and many kinds of natural focal diseases are distributed throughout Yunnan Province. Among these natural focal diseases, the harm of HFRS is very prominent. Except Diqing Tibetan Autonomous Prefecture, Lincang City and Dai Autonomous Prefecture of Xishuangbanna/Sipsongpanna, the other regions in Yunnan Province have all reported HFRS cases. In recent years, the old epidemic areas and foci in Yunnan Province are existing stably, and the HFRS cases in some areas have even increased significantly. Moreover, the newly discovered epidemic foci are increasing year by year, and the scope of the epidemic area is expanding [20]. The rich biological diversity and suitable natural geographical conditions of Yunnan Province have made it become a natural hotbed for the transmission and evolution of HV. With the rapid development of the tourism economy of Yunnan Province and the mobility of the population improving, which enhance the contact opportunities between the population and the virus host and disease transmission media, the risk of population exposure and the risk of population contracting diseases are increased [14]. In the residential areas and their surrounding environments, it is very likely to form an HFRS house-mouse-type epidemic area, posing a great threat to human health. Thus, it is necessary to carry out epidemiological and etiological studies of HFRS in various regions of Yunnan Province.

### 4.1. Expansion of the Monitoring Scope of Host Animal Species

In this paper, we describe a survey on the existence of SEOV in three counties/cities in Yunnan Province in Southern China in 2019. We found that the monitoring of dominant species should be paid attention to first. It is also worth noting that HV was also detected in *Rattus nitidus* this time, which is not the dominant species in Lianghe County, and the ratio of *Rattus nitidus* in the positive specimens is the same as that of *Suncus murinus*. In terms of the overall local capture quantity, *Rattus nitidus* only accounts for 0.86% of the total capture quantity in Lianghe County and belongs to the local rare species, which indicates that HV spillover can occur among various murine species even though the murine species is only a rare species in the local area. At the same time, among the many rare species in Lianghe County, *Rattus nitidus* is the most susceptible to infecting and carrying HV. Therefore, we should focus on the detection of HV in *Rattus nitidus* in Lianghe County.

This study elucidated that the detected positive rate of HV is the highest in the host animals in Lianghe County. Looking back on previous studies, it is not difficult to find that there were 91 pestis epidemics in Lianghe County from 1990 to 2006 [21]. The data indicate that among the 55 natural villages in Lianghe County, each village has had at least one pestis epidemic [22]. Moreover, as listed in Table 3, we compared a recent survey, which is based on the same time period, the same sampling point and the same animal specimen information as this study. This recent survey found that the infection rate of hepatitis E virus (HEV) among the murine-shaped animals in Lianghe County is also higher than those in the other two cities [23]. The higher infection rates of HV and HEV among the murine-shaped animals in Lianghe County suggest that the murine-shaped animals in the residential area and its surrounding areas in Lianghe County have a strong ability to infect and carry pathogens. It may also be that the landform, temperature, climate and cultural environment in the area of Lianghe County are very suitable for the propagation of various pathogens. Consequently, the pathogens carried by murine-shaped animals in Lianghe County, especially the double infection and multiple inflection, need to be emphatically monitored [24]. The monitoring of pathogens in Lianghe County may be the entry point for studying the high incidence mechanism of local pestis, and also is the key to discovering the high infection rate of HV and other pathogens in the local murine-shaped animals, which have great significance for the diagnosis and differential diagnosis of HV and the prevention and control of local HFRS.

In addition, Yunnan Province is a border province of China and borders Myanmar, Vietnam and Laos. Meanwhile, Yunnan Province is the economic and trade exchange center of Southeast Asian countries and maintains very close ties with Southeast Asian counties. For the prevention and control of HV, special attention should be paid to the spillover of overseas HV between the host animals of the two countries and the variability of genotype caused by the overflow among species. Many studies have shown that the host types of HV are extremely diverse [14,25,26,27]. It is necessary to expand the detection range of host animal species and pay attention to the monitoring of the virus-carrying rate in various small mammals, rather than only in *rodentia*.

### 4.2. Focusing on Genetic Variation

Based on the obtained results of genetic identification and phylogenetic analysis, it is shown that the HV of YNLH-K40 and YNLH-K53 found in Yunnan Province in this study is closest to the S5 subtype of the GOUV branch isolated from Longquan City, Zhejiang Province, China, and these subtypes are different from the main epidemic subtypes in Yunnan Province reported by previous studies. The published research has indicated that SEOV in Yunnan Province is dominated by S1 and S3 subtypes, and the host animals of SEOV in Yunnan Province are mainly *Rattus norvegicus* and *Rattus tanezumi* [17]. For the GOUV in Longquan City, Zhejiang Province, China, the host animals are also mainly *Rattus norvegicus* and *Rattus tanezumi* [10]. Thus, it is speculated that the HV found in *Rattus nitidus* in this study cannot be ruled out as the result of greater variation after overflow from the host animal. These findings suggest the genetic diversity of HV prevalent in some areas of Yunnan Province. As pointed out by Wang et al. [28], GOUV can cause human infection and is the main cause of HFRS in Longquan City, Zhejiang Province, China. Hence, it is necessary to further study the etiology of HV newly discovered in Lianghe County and especially pay attention to its infection and pathogenic potential.

Numerous data also prove that the genotype diversity of HV in Yunnan Province may be more abundant than expected [29,30,31,32]. In 2010, a new HV was discovered in *Eothenomys miletus* in Luxi County, Yunnan Province, named Luxi virus (LUXV) [29]. In 2012, a new virus similar to Luxi virus was found in *Microtus fortis* in Fugong County, Yunnan Province, named Fugong virus (FUGV) [30]. In 2006, Tula virus-like nucleic acid sequences were detected in the lung tissues of *Eothenomys miletus* in Luxi County, Yunnan Province [31]. Tula virus is originally distributed in Europe, and its main host animal is *Microtus arvalis*. Tula virus has proved that it can also cause the prevalence of HFRS [33]. Moreover, in 2016, a new HV, named DodeHV, was discovered in bats in the Pu’er area in Yunnan Province [32]. Sequence analysis exhibited that DodeHV has the highest homology with the XSV-VN1982B4 strain found in Phu thong Province of Vietnam in 2013, which further demonstrates that bats in border areas of China carry HV and have the risk of transmitting HV. Meanwhile, the high similarity between YNLH-K40 and YNLH-K53 and other subtypes of SEOV also reflects indirectly the frequent exchanges between regions promotes SEOV spread, which is consistent with the investigation results of Plyusnina et al. [34]. That is to say, many kinds of hosts carrying HV will accelerate the migration of HV under the convenient traffic circulation, thus quickening the transmission of SEOV in Yunnan Province, other regions of China and even abroad. This result further supports the recent hypothesis that the current global distribution for SEOV is attributed to the migration of Norway rats (*Rattus norvegicus*) [35].

In addition, the data analysis manifests that the amino acid homology of the obtained S-segment sequences YNLH-K40 and YNLH-K53 with SEOV is significantly higher than those with other types. Although the amino acid homology of YNLH-K40 and YNLH-K53 sequences with each subtype of SEOV is 83.6–87.8%, there is still a larger variation. For gene expression, each amino acid mutation may affect the function of the protein and thus influence the gene expression [36]. The S-segment gene of HV encodes nucleocapsid protein (NP). For HV, NP is the most abundant viral protein and is synthesized in the early stage of infection [37]. The S-segment has strong antigenicity and immunogenicity which can stimulate the cellular and humoral immune responses of the body, and the changes of HV self-characteristics are often related to the genetic variation in the S-segment [38,39]. At present, the S-segment gene can be used for the research and development of genetic engineering vaccines and nucleic acid vaccines [40]. Taking a vaccine is an extremely efficient and safe method for preventing HV infection, but the greater variation in amino acids will lead to the more likely failure of vaccine protection. Hence, more HV types and their variations should be systematically detected and focused on.

Although the SEOV carried by the *Rattus nitidus* in Lianghe County, Yunnan Province, has obvious regional characteristics, this investigation only collected the murine lung specimens in 2019 for HV detection, which has certain limitations. Given the evolutionary characteristics of selective host conversion and regional adaption of HV in natural transmission, it is necessary to strengthen the in-depth study on the host animal monitoring of HV and its genetic evolution features in the future. More importantly, it is needed to clarify the molecular epidemiology and pathogenicity properties of HV epidemic strains, which will play a positive role in the division, evolution and treatment of epidemic foci and the prevention and control of disease and will also be of great significance to the development of an HFRS vaccine and the clinical treatment.

## 5. Conclusions

In summary, the epidemic foci of HFRS in Yunnan Province are expanding year by year, and new hosts and new HV types are constantly being discovered. The situation of epidemic prevention and control cannot be ignored. In addition, nucleotide and amino acid variations may also invalidate existing vaccines. Thereby, it is very important to further monitor and discover the epidemic trend and variation law of various host animals and their HV infection, improve the establishment of the virus gene bank and pay attention to the research on the pathogenicity potential of new types of HV infection.

## Figures and Tables

**Table 1:** Detection of HV in murine-shaped animals in Yunnan Province, China.

Species	Region (Positive/Total)			Total (Positive/Total)
	Mile	Mangshi	Lianghe	
*Rattus tanezumi*	1/29 (3.45% ^a^)	2/100 (2.00% ^a^)	5/115 (4.35% ^a^)	8/244 (3.28% ^a^)
*Suncus murinus*	0/5	1/71 (1.41% ^a^)	1/74 (1.35% ^a^)	2/150 (1.33% ^a^)
*Rattus rattus/sladeni*	0/3	0/4	0/7	0/14
*Mus pahari*	0/5	0/1	0/7	0/13
*Tupaia belangeri*	0/5	0/3	0/5	0/13
*Hylomys suillus*	0	0/1	0/11	0/12
*Mus caroli*	0/11	0/1	0	0/12
*Crocidura attenuate*	0/4	0/1	0/5	0/10
*Rattus nitidus*	0/4	0	2/2 (100% ^a^)	2/6 (33.33% ^a^)
*Niviventer confucianus*	0	0	0/4	0/4
*Niviventer fulvescens*	0	0/3	0	0/3
*Crocidura horsfieldi tadae*	0	0/1	0/2	0/3
*Dremomys rufigenis*	0	0/1	0	0/1
*Tamiops swinhoei*	0	0/1	0	0/1
*Squirrel*	0	0/1	0	0/1
*Berylmys bowersi*	0	0/1	0	0/1
Total	1/66 (1.52% ^a^)	3/190 (1.58% ^a^)	8/232 (3.45% ^a^)	12/488 (2.46% ^a^)

Note: ^a^ represents the positive detection rate.

**Table 2:** Comparison of nucleotide and amino acid homologies of HV in Yunnan Province with other genotypes and subtypes.

Virus Strain	Type	1	2	3	4	5	6	7	8	9	10	11	12	13
1. YNLH-K40	Unclassified		99.3	86.0	86.5	84.3	86.7	88.5	85.2	66.3	41.0	41.1	65.5	48.1
2. YNLH-K53	Unclassified	99.3		85.9	86.5	84.0	86.8	88.5	85.3	65.7	40.6	40.2	66.0	48.0
3. AF288643	SEOV-S1	85.3	84.9		96.6	96.2	96.2	88.8	95.3	70.8	64.1	48.7	70.9	49.1
4. GU592948	SEOV-S2	85.5	85.0	97.2		96.3	96.4	89.3	95.7	71.2	63.9	48.4	70.7	53.8
5. AY766368	SEOV-S3	84.1	83.6	95.9	95.9		96.1	88.4	95.0	70.5	63.1	49.5	70.5	54.3
6. AY273791	SEOV-S4	85.2	84.7	96.5	96.5	95.7		88.8	96.0	71.5	62.7	49.7	71.5	53.7
7. JQ912777	SEOV-S5	87.8	87.3	88.1	88.7	87.0	88.4		88.2	70.8	62.9	49.1	71.1	54.0
8. AF329388	SEOV-S6	84.4	83.9	95.8	96.4	95.4	96.4	88.3		71.2	63.7	48.6	70.8	53.3
9. M14626	HTNV	70.0	70.0	72.8	72.9	72.5	73.0	71.9	73.2		60.8	50.4	72.2	53.0
10. L11347	PUUV	65.2	65.0	65.2	65.6	64.1	64.8	65.5	64.9	60.4		68.7	61.8	68.0
11. AF324902	ANDV	65.4	64.7	63.4	63.1	64.0	64.4	65.1	63.7	64.6	66.4		51.1	54.8
12. L41916	DOBV	70.7	70.3	72.2	71.8	71.8	73.3	71.6	72.5	73.2	62.4	65.6		50.7
13. L37904	SNV	63.8	64.4	65.8	65.1	64.1	65.4	65.5	64.5	63.9	66.5	74.2	63.9	

Note: the upper triangle part is the nucleotide sequence homology, and the lower triangle part is the homology of amino acid sequence.

**Table 3:** The infection rates of HEV and HV among murine-shaped animals in Yunnan Province, China.

Virus	Region			Reference
	Mile	Mangshi	Lianghe	
HEV	0.00%	4.69%	6.47%	Zhang [23]
HV	1.52%	1.58%	3.45%	This study

## Data Availability

YNLH-K40 and YNLH-K53 sequences were deposited in GenBank (https://www.ncbi.nlm.nih.gov/genbank) with accession numbers “OP381186-OP381187”.
